# The role of Rezum in the management of refractory urinary retention due to benign prostate hyperplasia: A literature review

**DOI:** 10.1080/2090598X.2023.2178104

**Published:** 2023-02-24

**Authors:** Ibrahim A. Khalil, Maya Aldeeb, Ahmed Mohammed, Khalid Awad, Tarek Ibrahim, Raed M. Al-Zoubi, Omar M Aboumarzouk, Khalid Al-Rumaihi

**Affiliations:** a Department of Urology, Hamad Medical Corporation, Doha, Qatar; b Department of Medical Education, Family Medicine Residency Program, Hamad Medical Corporation, Doha, Qatar; c Department of Surgery, Surgical Research Section, Hamad Medical Hospital, Hamad Medical Corporation, Doha, Qatar; d Department of Biomedical Sciences, College of Health Sciences, QU-Health, Qatar University, Doha, Qatar; e Department of Chemistry, Jordan University of Science and Technology, Irbid, Jordan; f College of Medicine, Qatar University, Doha, Qatar; g Veterinary and Life Science, The University of Medicine, University of Glasgow, Scotland, UK

**Keywords:** Benign prostate hyperplasia, refractory retention, Rezum, TURP

## Abstract

**Background:**

Benign prostatic hyperplasia is the most common cause of urinary retention in men (BPH). The gold standard surgical treatment is transurethral resection of the prostate (TURP). However, due to the morbidity and mortality associated with TURP, more minimally invasive treatments, such as vaporizing the prostate with the Rezum system, have been introduced. We investigated the efficacy of Rezum in the treatment of refractory urinary retention due to BPH in this review.

**Methodology and materials:**

To conduct this review, the Cochrane methodology for systematic reviews was used. All studies that used Rezum to treat catheter-dependent patients with enlarged prostates were included. The literature search showed 111 studies, 84 of which were excluded due to non-relevance based on titles and 18 due to lack of relevance based on abstract review. Full manuscripts were reviewed in nine studies, three of which were excluded because they did not meet the inclusion criteria.

**Results:**

This review included 301 patients in total. The rate of a successful trial of voiding post Rezum therapy was 85%. The complication rated between 3.8 and 4.3% all of which were mild and self-limited. As there was no major complication of Rezum (clavien dindo >2), the procedure-related morbidity is negligible.

**Conclusion:**

In this review, Rezum was found to be an efficacious and safe alternative in the treatment of refractory retention with mild complications and minimal morbidity.

## Introduction

Benign prostatic hyperplasia (BPH) is the most common cause of voiding and lower urinary tract symptoms in men, particularly in men over the age of 60 [1,2]. Patients who present with urinary retention are initially fitted with a urethral catheter and start medical treatment, principally alpha blockers, followed by a voiding trial; failure to void despite medical treatment is considered refractory retention, which is an absolute indication for surgical intervention in the management of BPH [3].

The gold standard surgical treatment for BPH remains transurethral resection of the prostate (TURP) [4]. However, morbidity and mortality of TURP along with the effects on sexual function and infertility necessitated the development of a more minimal invasive intervention such as Aquablation, Prostatic urethral lift (Urolift), and Rezum systems [5–7].

The Rezum System ablates prostatic tissue using convective water vapor energy and can be performed as an office-based procedure with local anaesthesia and oral analgesia to avoid the morbidities associated with general or even spinal anaesthesia required in TURP. The efficacy and safety of Rezum system have been reported in multiple prospective and retrospective studies as efficacy was associated with low rate of shortlived mild side effects [8–10]. Due to its low complication profile and efficacy, Rezum is a promising intervention for BPH, especially in patients with multiple comorbidities who cannot tolerate TURP or in patients who are concerned about fertility and sexual function complication of TURP.

Several studies looked into the effectiveness of Rezum in treating lower urinary tract symptoms caused by BPH, but the role of Rezum in the treatment of refractory retention remains uncertain. In this review, we evaluated the efficacy of Rezum in treating patients with refractory urinary retention due to benign prostate hyperplasia as an alternative to TURP.

## Methodology and materials

### Search strategy

To conduct this review, the Cochrane methodology for systematic reviews was used [11,12]. The risk of bias within each study was assessed using the ROBINSI tool to ensure the validity and reliability of the retrospective studies included in this paper [13]. Google Scholar, the US National Library of Medicine’s life science database (MEDLINE), and individual recognized urology journals were used in the search strategy. ‘Rezum’ and ‘urinary retention’ were two search terms that were used together. (Rezum[All Fields] AND (‘urinary retention’[MeSH Terms] OR (‘urinary’[All Fields] AND ‘retention’[All Fields]) OR ‘urinary retention’[All Fields]) AND (‘2016/1/1’[PubDate]: ‘3000’[PubDate]) AND (‘2016/1/1’[PubDate]: ‘3000’[PubDate]).

### Study selection and data extraction

We included all studies that reported on the use of Rezum in the treatment of enlarged prostate patients with chronic retention and catheter-dependent patients. Two authors independently identified studies that were eligible for inclusion and extracted the data. The authors’ disagreement was settled by unanimous agreement. Patient demographics, prostate size, a successful voiding trial, and catheter-free rate were among the variables extracted for the patient with retention.

## Results

### Literature search

**Figure 1.**
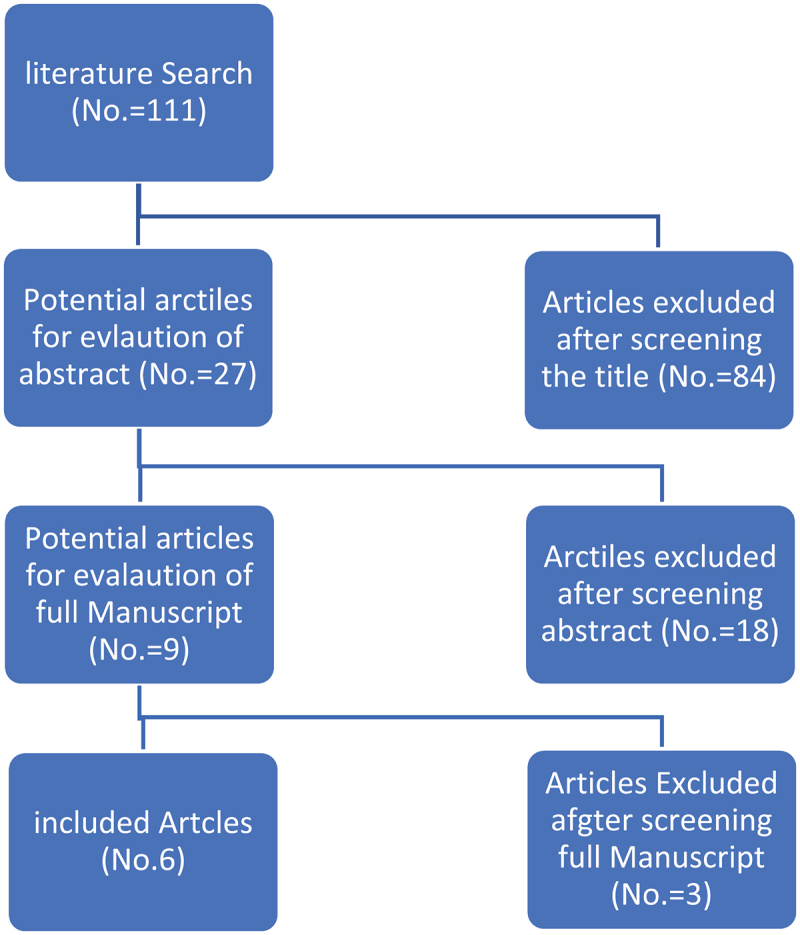
Flow chart for articles selection process of the review.

The literature search showed 111 studies, of which 84 were eliminated due to non-relevance based on titles and 18 were eliminated due to lack of relevance based on abstract review (Figure 1). Full manuscripts were reviewed in nine studies, three of which were excluded because they did not meet the inclusion criteria.

### Inclusion criteria

The inclusion criteria articles that describe the use of Rezum in the treatment of refractory urinary retention. Articles that do not have data regarding patients with retention were excluded.

### Characteristics of the included studies

The studies spanned between Nov 2016 and April 2022. The included studies evaluated the efficacy of Rezum in managing enlarged prostate. The number of patients in the included articles ranged from 8 to 136 patients. All the included studies found to have low risk of bias on ROBINS-I tool.

### Patient demographics

This study included 301 patients in total; all of whom had urinary retention due to enlarged prostate and were catheter-dependent; the mean age was 75.7 years (59 − 89). Mean prostate volume was 74(20–200) cc. The mean preoperative retention volume of 888(566–2100) cc was reported in three articles involving 195 patients (Table 1).

**Table 1.** The included studies, patients demographics and outcomes after Rezum treatment for BPH with chronic urinary retention.

Author	Number of patients	Mean age (years)	ASA classification	Mean prostate volume (cc)	Catheter-free rate	Mean IPSS score post Rezum	Mean IPSS Qol post Rezum
Bole et al^[^14^]^	60	70.3	––––	84	85%	––––*	––––
Bassily et al^[^15^]^	49	73	3 (2,3) (IQR)	73	88%	4	1
Aleogorides etal^[^16^]^	8	74.6	––––	71.8	100%	5.8	1.2
Eredics et al^[^17^]^	136	80.3	Grade II: 10% Grade III:71% Grade IV: 19%	54	78.6%	––––	––––
Wong et al^[^18^]^	10	74.9	––––	73.8	100%	4.5	0.7
McVary et al^[^19^]^	38	75.5	––––	58.5	70.3%	10.5	2.1

Abbreviations: ASA, American Society of Anaesthetists; IQR, interquartile range;IPSS: International Prostatism Symptom Score, Qol: quality of life, * Raveti et al used American Urological Association Symptom.

### Intervention and outcome

All patients in this review underwent Rezum procedure; the median number of vapor injections used was 10 [5–12] [14–19].

The success rate of a voiding post Rezum therapy trial was 85%, as 256 out of 301 patients were catheter free after the procedure, with follow-up periods ranging between 3 and 12 months. Two articles subclassified patients according to prostate volume to < 80 ccs and > 80 ccs, the catheter-free rate of those groups was 83% and 77.5%, respectively.

The outcome of patients who failed TWOC was reported in 2 articles, 10 patients had reintervention, 3 of them underwent transurethral resection of prostate; 2 of them failed TWOC after it; another 3 patients underwent redo Rezum; and all of them failed TWOC. Four of them remained catheter-dependent either SPC or CIC.

Post-Rezum PVR was 144 ccs, and a post Rezum Q max of 11.6 ml/sec has reported in three studies. The post-Rezum improvement of lower urinary tract symptoms reported in five studies, the mean post-Rezum IPSS was 6.2 (SD+-2.9} in four studies, while one study used the AUA symptoms score, which was 12.75 post-Rezum.

The reported complications were mild in nature with clavien dindo grade I treated conservatively. The complication was reported in four studies involving 233 patients; in which, 10 (4.3%) patients had gross haematuria and 9 (3.8%) patients had urinary tract infection; no other complications were reported.

## Discussion

The most common cause of urinary retention in men is BPH; the incidence increases with age where men aged 70 and above are at higher risk [6,20]. The decision between catheterization, surgical intervention for chronic and refractory is challenging. The studies showed the favourable outcome of TURP compared to catheterization in terms of quality of life, and the rate of urinary tract infections [21].

In this review, we studied the Rezum system’s efficacy in refractory urinary retention and found that the catheter-free rate post-Rezum is 85% (number of studies 6 and number of patients 301). The most important factor affecting the rate of successful TWOC was prostate volume, as prostate volume > 80 ccs was associated with lower catheter-free rates (number of studies 2 and number of patients 196). The catheter-free rate of the gold-started transurethral resection of the prostate in the treatment of BPH-induced urinary retention is 92% [22], which is comparable to the results of Rezum in our review (85%). The higher catheter-free rate comes with the expense of higher morbidity and mortality as TURP has a higher incidence of bleeding, incontinence, erectile dysfunction, and retrograde ejaculation compared to Rezum [23].

The reported suboptimal Q max and PVR post-Rezum in patients with refractory retention might be a result of an element of decreased bladder contractility as the patients who failed to void without catheter after Rezum also failed to void after going TURP or redo Rezum and remained catheter-dependent [15,17,24]. In addition, the preoperative risk factors affecting voiding function such as decreased bladder contractility has been reflected in comparable retreatment rates between Rezum and TURP (4.76%–8.33% and 3%–14.5%, respectively) [24–26]. Those findings that emphasize the importance of proper evaluation of the bladder contractility are the cases of failed treatment to avoid unnecessary reinterventions and to predict the outcome.

The effect on sexual function is a major determinant in the management of BPH, for instance the gold standard surgical treatment TURP results in retrograde ejaculation in 50–70% of patients [27]. On the other hand, alpha blocker therapy is associated with reduced ejaculate volume in 90% of patients and anejaculation in 35% of patients. [28]. While the rate of retrograde ejaculation of Rezum is as low as 2% of patients [29]. In our review, the studies showed that erectile function was not affected by Rezum treatment [17,18]. This minimal effect of Rezum on the overall sexual function when compared to TURP makes Rezum superior in terms of preservation of sexual function and fertility.

The adverse effects of Rezum are related to endoscopic instrumentation during the procedure. When compared to TURP, Rezum complications are mild and self-resolving that includes haematuria (11.8%), dysuria (16.9%), urgency (5.9%), frequency and acute urinary retention (3.7%) and urinary tract infection (3.7%) [10,18] . On the other hand, TURP is associated to be more severe that might be life-threatening complications. TURP complication includes but not limited to significant bleeding (0.4%–7.1%), clot retention (2%–5%), electrolyte imbalance and TUR syndrome (0.0%–1.1%), urinary tract infection (1.7%−8.2%) and urinary retention (3% – 9%). Also complications of TURP can be delayed and present in the picture of bladder neck contractures and urethral strictures in about 0.3–9.8% of the cases [25]. While no mortality was reported as post-Rezum, the TURP mortality rate ranges between 0 and 0.25% and increases with age and comorbidities [6,25].

In this review, we observed that Rezum is a safe and effective alternative to TURP in the treatment of refractory urinary retention due to BPH. The main limitation of this review is the low quality of the studies included, as the majority of the studies were retrospective and had a relatively short follow-up period. In addition, some studies included patients with refractory urinary retention as a subgroup.

## Conclusion

In this review, Rezum was found to be an efficacious and safe alternative in the treatment of refractory retention with mild complications and minimal morbidity. More research is needed to prove the efficacy of Rezum as an alternative to TURP and to evaluate its long-term complications.
